# Cost of a human papillomavirus vaccination project, Zimbabwe

**DOI:** 10.2471/BLT.18.211904

**Published:** 2018-10-17

**Authors:** Anna Hidle, Gwati Gwati, Taiwo Abimbola, Sarah W Pallas, Terri Hyde, Amos Petu, Deborah McFarland, Portia Manangazira

**Affiliations:** a United States Centers for Disease Control and Prevention, 1600 Clifton Road, Mail Stop H24-2, Atlanta Georgia, 30329, United States of America (USA).; b Ministry of Health and Child Care, Government of Zimbabwe, Harare, Zimbabwe.; c Immunization Financing Sustainability, InterCountry Support Team, East & Southern Africa, World Health Organization, Harare, Zimbabwe.; d Rollins School of Public Health, Emory University, Atlanta, USA.

## Abstract

**Objective:**

To determine the cost of Zimbabwe’s human papillomavirus (HPV) vaccination demonstration project.

**Methods:**

The government of Zimbabwe conducted the project from 2014–2015, delivering two doses of HPV vaccine to 10-year-old girls in two districts. School delivery was the primary vaccination strategy, with health facilities and outreach as secondary strategies. A retrospective cost analysis was conducted from the provider perspective. Financial costs (government expenditure) and economic costs (financial plus the value of existing or donated resources including vaccines) were calculated by activity, per dose and per fully immunized girl.

**Results:**

The project delivered 11 599 vaccine doses, resulting in 5724 fully immunized girls (5540 at schools, 168 at health facilities and 16 at outreach points). The financial cost for service delivery per fully immunized girl was United States dollars (US$) 5.34 in schools, US$ 34.90 at health facilities and US$ 288.63 at outreach; the economic costs were US$ 17.39, US$ 41.25 and US$ 635.84, respectively. The mean financial cost per dose was US$ 19.76 and per fully immunized girl was US$ 40.03 (economic costs were US$ 45.00 and US$ 91.19, respectively). The largest number of doses delivered (5788) occurred during the second vaccination round (the second group’s first dose concurrently delivered with the first group’s second dose), resulting in the lowest financial and economic service delivery costs per dose: US$ 1.97 and US$ 6.79, respectively.

**Conclusion:**

The mean service delivery cost was lower in schools (primary strategy) and when more girls were vaccinated in each round, demonstrating scale efficiency.

## Résumé

**Objectif:**

Déterminer le coût du projet de démonstration de la vaccination contre le papillomavirus humain (PVH) au Zimbabwe.

**Méthodes:**

Le gouvernement du Zimbabwe a mené ce projet de 2014 à 2015, en administrant deux doses de vaccin contre le PVH aux filles de 10 ans de deux districts. La stratégie de vaccination principale a consisté à intervenir dans les écoles, puis dans des établissements de santé et des centres de proximité comme stratégies secondaires. Une analyse rétrospective des coûts a été effectuée du point de vue des prestataires. Les coûts financiers (dépenses du gouvernement) ainsi que les coûts économiques (coûts financiers plus valeur des ressources existantes ou mises à disposition, y compris les vaccins) ont été calculés par activité, par dose et par fille totalement immunisée.

**Résultats:**

Le projet a permis d'administrer 11 599 doses de vaccins et d'immuniser totalement 5724 filles (5540 à l'école, 168 dans des établissements de santé et 16 dans des centres de proximité). Le coût financier de la prestation de services par fille totalement immunisée était de 5,34 dollars des États-Unis ($ US) pour la vaccination réalisée à l'école, de 34,90 $ US dans des établissements de santé et de 288,63 $ US dans des centres de proximité; les coûts économiques étaient respectivement de 17,39 $ US, 41,25 $ US et 635,84 $ US. Le coût financier moyen était de 19,76 $ US par dose et de 40,03 $ US par fille totalement immunisée (les coûts économiques étaient respectivement de 45,00 $ US et de 91,19 $ US). Le plus grand nombre de doses (5788) a été administré durant la deuxième série de vaccinations (première dose du second groupe administrée en même temps que la deuxième dose du premier groupe), où ont été relevés les plus faibles coûts financiers et économiques de la prestation de services par dose: respectivement 1,97 $ US et 6,79 $ US.

**Conclusion:**

Le coût moyen de la prestation de services était plus faible lors de la vaccination dans les écoles (stratégie principale) et lorsque l'on vaccinait un grand nombre de filles dans chaque série de vaccinations, ce qui révèle des économies d'échelle.

## Resumen

**Objetivo:**

Determinar el coste del proyecto de demostración de vacunación contra el virus del papiloma humano (VPH) en Zimbabwe.

**Métodos:**

El gobierno de Zimbabwe llevó a cabo el proyecto entre 2014 y 2015, administrando dos dosis de la vacuna contra el VPH a niñas de 10 años en dos distritos diferentes. La estrategia de vacunación primaria fue la administración de la misma en colegios, con los centros sanitarios y la difusión como estrategias secundarias. Se realizó un análisis retrospectivo de los costes desde la perspectiva del proveedor. Los costes financieros (gasto público) y los costes económicos (los costes financieros más el valor de los recursos existentes o donados, incluidas las vacunas) se calcularon por actividad, por dosis y por niña totalmente inmunizada.

**Resultados:**

El proyecto administró 11 599 dosis de la vacuna, lo que dio como resultado 5724 niñas totalmente inmunizadas (5540 en los colegios, 168 en los centros sanitarios y 16 en los puntos de difusión). El coste financiero de la prestación de servicios por niña totalmente inmunizada fue de 5,34 dólares estadounidenses en los colegios, 34,90 USD en los centros de salud y 288,63 USD en las actividades de difusión; los costes económicos fueron de 17,39 USD, 41,25 USD y 635,84 USD, respectivamente. El coste financiero medio por dosis fue de 19,76 dólares estadounidenses y por niña totalmente inmunizada de 40,03 USD (los costes económicos fueron de 45,00 USD y 91,19 USD, respectivamente). El mayor número de dosis administradas (5788) se produjo durante la segunda ronda de vacunación (la primera dosis del segundo grupo administrada simultáneamente con la segunda dosis del primer grupo), lo que dio lugar a menores costes financieros y económicos de prestación de servicios por dosis: 1,97 USD y 6,79 USD, respectivamente.

**Conclusión:**

El coste medio de la prestación de servicios fue menor en los colegios (estrategia primaria) y cuando se vacunó a más niñas en cada ronda, lo que demuestra la eficiencia de la escala.

## ملخص

**الغرض:**

تحديد تكلفة المشروع التوضيحي للتطعيم (HPV) ضد فيروس الورم الحليمي البشري في زمبابوي.

**الطريقة:**

قامت حكومة زيمبابوي بتنفيذ المشروع خلال الفترة من 2014 إلى 2015، حيث قدمت جرعتين من لقاح فيروس الورم الحليمي البشري للفتيات في سن العاشرة من العمر في مقاطعتين. كان تقديم التطعيم في المدرسة هو إستراتيجية التطعيم الأولية، حيث كانت المرافق الصحية والتوعية استراتيجيات ثانوية. تم إجراء تحليل للتكاليف بأثر رجعي من وجهة نظر مقدم الخدمة. تم احتساب التكاليف المالية (النفقات الحكومية) والتكاليف الاقتصادية (المالية بالإضافة إلى قيمة الموارد الحالية أو التي تم التبرع بها بما في ذلك اللقاحات) حسب النشاط ، لكل جرعة ولكل فتاة محصنة بالكامل.

**النتائج:**

قدم المشروع 11599 جرعة من اللقاح، مما نتج عنه 5724 فتاة محصنة بالكامل (5540 في المدارس، و168 في المرافق الصحية، و16 في مراكز التوعية). وبلغت التكلفة المالية لتقديم الخدمات لكل فتاة محصّنة كلياً 5.34 دولاراً أمريكياً في المدارس، و34.90 دولاراً أمريكياً في المرافق الصحية، و288.63 دولاراً أمريكياً في مجال التوعية؛ وبلغت التكاليف الاقتصادية 17.39 دولاراً أمريكياً، و41.25 دولاراً أمريكياً، و635.84 دولار أمريكي على التوالي. بلغ متوسط التكلفة المالية لكل جرعة 19.76 دولاراً أمريكياً، ولكل فتاة محصّنة بالكامل 40.03 دولاراً أمريكياً (كانت التكاليف الاقتصادية 45.00 دولاراً أمريكياً و91.19 دولاراً أمريكياً على التوالي). كان أكبر عدد من الجرعات التي تم تسليمها (5788) خلال الجولة الثانية من التطعيم (الجرعة الأولى للمجموعة الثانية تم تسليمها في وقت متزامن مع الجرعة الثانية للمجموعة الأولى)، مما أدى إلى انخفاض تكاليف تقديم الخدمات المالية والاقتصادية لأقصى حد لكل جرعة: 1.97 دولار أمريكي و6.79 دولاراً أمريكياً على التوالي.

**الاستنتاج:**

كان متوسط تكلفة تقديم الخدمة أقل في المدارس (الاستراتيجية الأولية)، وعندما تم تلقيح المزيد من الفتيات في كل جولة، مما يدل على كفاءة المقياس.

## 摘要

**目的:**

确定津巴布韦人乳头瘤病毒 (HPV) 疫苗接种示范项目的成本。

**方法:**

津巴布韦政府于 2014 年至 2015 年进行该项目，在两个地区向 10 岁的女孩提供了两剂 HPV 疫苗接种。基于学校的疫苗接种是主要的疫苗接种战略，基于医疗机构和外展疫苗接种作为次要战略。从提供者的角度进行回顾性成本分析。财政成本（政府支出）和经济成本（财政加上现有的或捐赠的资源，包括疫苗）是根据活动、单位剂量和每名获得完全免疫的女孩计算得出。

**结果:**

该项目提供了 11599 剂疫苗，令 5724 名女孩获得了完全免疫 （5540 名通过学校的疫苗接种，168 名通过医疗机构的疫苗接种，另外 16 名通过外展疫苗接种点进行接种）。每名获得完全免疫的女孩所需的财务成本为：基于学校的疫苗接种为 5.34 美元 ，基于医疗机构的疫苗接种为 34.90 美元，通过外展疫苗接种点的疫苗接种为 288.63 美元；经济成本分别为 17.39 美元、41.25 美元和 635.84 美元。每剂疫苗的平均财务成本为 19.76 美元，每名获得完全免疫的女孩的平均财务成本为 40.03 美元（经济成本分别为 45.00 美元和 91.19 美元）。第二次疫苗接种（第二组的第一剂疫苗与第一组的第二剂疫苗同时接种）期间接种的剂量最多 （5788 剂），导致每剂接种的财政和经济服务成本最低：分别为 1.97 美元和 6.79 美元。

**结论:**

基于学校进行疫苗接种的平均服务接种成本较低（主要战略），而且每一轮进行疫苗接种的女孩数量越多，规模效益越显著。

## Резюме

**Цель:**

Определение затрат на демонстрационный проект вакцинации против вируса папилломы человека (ВПЧ) в Зимбабве.

**Методы:**

Правительство Зимбабве осуществляло проект с 2014–2015 гг., вводя по две дозы вакцины против ВПЧ 10-летним девочкам в двух районах страны. Основная стратегия распределения вакцины подразумевала участие школ, а учреждения здравоохранения и пропаганда вакцинации рассматривались как стратегии второго порядка. Был проведен ретроспективный анализ затрат с точки зрения поставщика услуг. Финансовые расходы (государственные расходы) и экономические издержки (финансовые расходы плюс стоимость имевшихся или пожертвованных ресурсов, включая вакцины) рассчитывались с разбивкой по роду деятельности из расчета на дозу и на одну полностью иммунизированную девочку.

**Результаты:**

В ходе проекта было поставлено 11 599 доз вакцины и были полностью иммунизированы 5724 девочки (5540 в школах, 168 в учреждениях здравоохранения и 16 в пунктах распространения информации). Финансовые расходы на оказание услуг из расчета на одну полностью иммунизированную девочку составили в долларах США: 5,34 в школах, 34,90 в учреждениях здравоохранения и 288,63 в пунктах распространения информации. Экономические издержки составили 17,39; 41,25 и 635,84 доллара США соответственно. Средний показатель финансовых расходов составил из расчета на дозу 19,76 доллара США и из расчета на одну полностью иммунизированную девочку 40,03 доллара США (соответственные экономические издержки составили 45,00 и 91,19 доллара США). Наибольшее количество доз (5788) было поставлено в ходе второго тура вакцинации (первая доза второй группы поставлялась одновременно со второй дозой первой группы), что позволило достичь минимальных финансовых и экономических затрат на оказание услуг из расчета на дозу: 1,97 и 6,79 доллара США соответственно.

**Вывод:**

Средняя стоимость оказания услуг была ниже для школ (основная стратегия) и в тех случаях, когда в каждом туре вакцинировалось большее количество девочек, что демонстрирует зависимость уровня эффективности от масштаба охвата.

## Introduction

Each year 266 000 women worldwide die of cervical cancer due to human papillomavirus (HPV) infection.^1^^,^^2^ Cervical cancer is the fourth leading cause of estimated cancer deaths worldwide among women, most of which occur in low- and middle-income countries; the total is projected to increase to 416 000 deaths by 2035.^1^^,^^3^^,^^4^ Low- and middle-income countries account for 84% (444 500 out of 527 600) of the world’s cervical cancer burden.^2^^,^^4^ HPV infection, one of the most common sexually transmitted diseases worldwide, is known as the main cause of cervical cancer, with HPV types 16 and 18 causing most cervical cancer cases.^5^ The World Health Organization (WHO) recommends that all countries add the HPV vaccine to their national immunization programme, selecting a delivery strategy that is feasible with the current health infrastructure, affordable, cost–effective, sustainable and capable of achieving high coverage.^6^

Zimbabwe is a low-income country with an estimated 4.9 million women aged 15 years and older at risk of developing cervical cancer.^5^^,^^7^^,^^8^ Cervical cancer is the most frequent cancer among women (2270 new estimated cases of 8997 cancer cases in women each year) and the leading cause of morbidity from all cancers in Zimbabwe.^2^ Zimbabwe’s national health strategy and cancer prevention and control strategies mention HPV vaccination as a way to avert cervical cancer.^9^^,^^10^

In 2013, Zimbabwe’s Ministry of Health and Child Care proposed an HPV vaccination demonstration project supported financially by Gavi, the Vaccine Alliance. Gavi support for HPV vaccination demonstration projects was intended to allow countries to better understand the strategies and costs to deliver vaccine to a target population of girls aged 9–13 years.^11^ Although Zimbabwe’s project was initially designed with a three-dose schedule, it was revised to provide a two-dose schedule following updated guidance issued by WHO in 2014.^12^ To date, only a few published studies of the costs of HPV vaccination demonstration projects exist.^13^^–^^16^ Our cost analysis adds original empirical retrospective data for delivering a two-dose vaccination schedule to two groups of girls in Zimbabwe, including the cost of vaccinating overlapping groups of girls (a second group’s first dose concurrently delivered with a first group’s second dose).

The existing literature on the cost of HPV vaccination demonstration projects has mostly focused on three-dose schedules and single cohorts.^13^^–^^16^ The aim of this study was to determine the cost of Zimbabwe’s two-dose HPV vaccination demonstration project. The study also contributes evidence on the costs of vaccinating overlapping groups of girls, providing insight into potential scale efficiencies. The study used a detailed empirical retrospective costing approach based on actual expenditure to understand the actual costs of delivering the vaccine.

## Methods

### Project implementation

The HPV vaccination project delivered vaccines to 10-year-old girls in two districts in 2014 and 2015 (Box 1). Zimbabwe selected school-based vaccination as the primary delivery strategy, with secondary delivery strategies at health facilities and outreach points (such as farms and missions) to reach girls not attending school or who were absent during school vaccinations. Social mobilization was led by two local nongovernmental organizations (NGOs) and health ministry vaccinators to identify girls who missed vaccination. School health coordinators (teachers from the school trained in aspects of health) and school head teachers assisted in the logistics. These teachers scheduled vaccination dates, verified the eligibility of girls for vaccination by confirming birth dates, ascertained the fitness of those eligible for vaccination, reported any adverse events following immunization and facilitated referrals to the clinic if necessary. In each district, village health workers were engaged to offer support during the vaccination rounds, while a command centre facilitated logistics, monitored data collection and relayed information to the national level.

Box 1 **Study setting of the Zimbabwe human papillomavirus vaccination project, 2014–2015**

**Selected districts**

Beitbridge district in Matabeleland South province (district population: 122 553)

Marondera district in Mashonaland East province (district population: 178 547)

**Selection criteria for districts**

Peri-urban (two-thirds rural) districts

Two major ethnic groups represented (Ndebele and Shona)

High diphtheria–tetanus–pertussis vaccination coverage: > 90%

Experience of schoolchildren vaccination programmes that would provide a potential platform for integrated delivery of HPV vaccine and other health interventions in the future

**Target age group**

10-year-old girls residing in the selected districts

**Target population for vaccination^a^**

Beitbridge district: 2628 (1700 first group, 928 second group)

Marondera district: 3880 (2043 first group, 1837 second group)

**HPV vaccine administered**

Bivalent vaccine

**Number of vaccine doses for a fully immunized girl**

Two doses

**Primary delivery strategy**

Primary schools

**Secondary delivery strategies**

Health facilities; outreach points

HPV: human papillomavirus.^a^ The method of enumeration, based on school registers, likely underestimated the number of out-of-school girls.Data sources: Government of Zimbabwe, 2013.^17^

Both the selected districts in Zimbabwe implemented the project to reach an initial group of girls using three vaccination rounds (Table 1). The target population for vaccination, based on school registers, was 2628 in Beitbridge district and 3880 in Marondera district. The first vaccination round attempted to reach the target population with the first dose. The second vaccination round delivered the second dose to the initial group of girls from the first vaccination round and the first dose to girls who missed the first vaccination round or who had recently turned 10 years old. A third vaccination round provided an opportunity to complete the vaccination series for girls from either of the previous rounds.

**Table 1:** Dosing schedule by vaccination round in two districts in the Zimbabwe human papillomavirus vaccination project, 2014–2015

Vaccination round	Aim	Group	Dose	Date of dose 1		Interval between dose 1 and dose 2, months	
				Beitbridge	Marondera	Beitbridge	Marondera
First	To deliver dose 1 to the target population of 10-year-old girls in two districts	1	1	Sep 2014	Sep 2014	NA	NA
Second	To deliver dose 2 to girls from the first vaccination round and dose 1 to girls who missed the first round or who had recently turned 10 years old	1, 2	2, 1	Jun 2015	Mar 2015	9	6
Third	To complete the vaccination series for girls from either of the previous rounds	2	2	Nov 2015	Nov 2015	6	8

NA: not applicable.

### Study design

We used a retrospective, ingredients-based approach to estimate the incremental, or additional, costs of implementing the HPV vaccination project. Costs were collected from the provider perspective, which included all costs of implementing the project from the government and donors, and were further categorized into financial and economic costs. Financial costs were defined as actual monetary payments or expenditure by the government, while economic costs were defined as financial costs plus the value of resources already paid for or owned by government or provided by other sources (opportunity cost), including the value of donated vaccines. We based costs on the resources (ingredients) used for each major activity as defined by the WHO Cervical Cancer Prevention and Control Costing tool user guide.^18^

Financial costs included expenditure by the health ministry.^18^^,^^19^ Economic costs included financial costs plus expenditure by partners (e.g. vaccine and vaccine procurement by United Nations Children’s Fund, funded by Gavi) and in-kind resource contributions from the health ministry and partners. We did not include evaluation costs for the coverage survey or cost analysis and external partner technical assistance, except for the economic costs of the post-introduction evaluation, according to the WHO Cervical Cancer Prevention and Control Costing tool user guide.^18^

For each activity, we calculated the value of the resources used based on actual expenditure, or the quantity of the resource used multiplied by that resource’s unit cost and then summed across all resources. We estimated the daily value of personnel time using annual salary plus benefits by cadre divided by 222 working days per year; volunteer time was valued using a minimum wage estimate provided by the health ministry.^18^^,^^19^ We valued in-kind vehicle donations at a mean cost of the vehicle type based on new sale price (assuming no resale value, a useful life of 5 years and 222 working days per year), multiplied by the days the vehicle was used for the project. The vaccine purchase price per dose was United States dollars (US$) 4.60 (US$ 9.20 for a 2-dose vial); for analysis, we used the price of US$ 5.06 per dose procured after adding a 10% charge for airport clearance and transportation to the central medical stores.^11^ Our cost analysis presents the empirical retrospective economic cost of vaccines actually used in the project, including doses delivered to the target population and doses wasted.

Since there were no major improvements made to the existing cold-chain infrastructure for the project (e.g. purchasing additional vaccine carriers or refrigerators), we calculated opportunity costs by using existing vaccine carriers and cold packs, as these were borrowed from routine vaccination sessions. We did not report the cost of vaccination waste disposal because the central medical stores and implementing districts were not able to provide an estimate of the share of waste disposal specific to HPV vaccination waste. Some aspects of service delivery costs (e.g. fuel) were reported by the health ministry as lump-sum costs. We divided these costs by the total number of vaccination sites reached in the vaccination round during which the expense was incurred to obtain cost per site, and then multiplied by the number of sites for each strategy (school, health facility or outreach) to obtain cost per strategy in the absence of information on resources used to reach each individual site.

We designated costs as introduction or recurrent costs based on the WHO Cervical Cancer Prevention and Control Costing tool user guide,^18^ to delineate one-time investments from costs expected to continue on a recurring basis beyond the vaccine introduction phase. We defined introduction costs as investments needed during the initial phase of introducing a new vaccine into the immunization programme (i.e. investments in cold chain; social mobilization and information materials; microplanning; and training of supervisors, vaccinators and school staff). We defined recurrent costs as costs expected to continue on an ongoing basis (i.e. procurement of vaccines and related supplies; service delivery; and supervision, monitoring and evaluation).^18^

This analysis considers the incremental resources needed to add HPV vaccination to an existing immunization programme. We excluded the costs of routine operation of the health system and immunization programme.^18^ We collected costs in current prices from 2014, 2015 and 2016, adjusted for inflation to 2016 US$ using the Zimbabwe consumer price index.^20^

### Data collection and analysis

We collected the data through in-person consultations and by phone and email from 25 individuals from NGOs and the health ministry at the national, provincial, district and health-facility levels. Follow-up consultations were held with the same individuals to clarify and validate the initial information. We reviewed relevant programmatic and financial documents (e.g. annual reports, work plans, programme budgets and Zimbabwe’s Gavi application^17^) to triangulate with interview data and use as secondary data sources.

All resources recommended by the WHO Cervical Cancer Prevention and Control Costing tool were included in the data collection design for financial and economic costs by type of activity. Activities for which costs were reported in Zimbabwe are presented in Table 2. We conducted data analysis using Excel software, version 16.0 (Microsoft Corp., Redmond, United States of America) using the WHO Cervical Cancer Prevention and Control Costing tool user guide.^18^ We estimated total financial and economic costs, then divided these by the number of doses and number of fully immunized girls to obtain the cost per dose and cost per fully immunized girl, respectively. We calculated introduction and recurrent costs using the cost of all doses delivered plus the cost of estimated doses wasted for groups in both districts. We calculated the service delivery category of costs separately by delivery strategy.

**Table 2:** Resource ingredients included in financial and economic cost calculations by activity category in the Zimbabwe human papillomavirus vaccination project. 2014–2015

Activity	Items included in financial costs^ a^	Additional items included in economic costs^b^
Microplanning	• Venue rental; • Per diem payments (lodging, meals); • Fuel and transportation fees; and • Other supplies and materials	• Venue rental; • Per diem payments (lodging, meals); • Fuel and transportation fees; • Other supplies and materials; and • Personnel time (share of salaries and benefits)
Vaccines	• Vaccination supplies (safety boxes, cotton)	• Vaccines; • Syringes; • Freight, customs, transport of vaccine and related supplies; and • Use of vaccine carriers and cold packs
Training	• Per diem payments (lodging, meals); • Fuel and transportation fees; and • Other supplies and materials	• Personnel time (share of salaries and benefits)
Social mobilization and information materials	• Per diem payments (lodging, meals); • Fuel and transportation fees; • Other supplies and materials; and • Personnel time (share of NGO contractor salaries and benefits)	• Fuel; • Other supplies and materials; • Vehicle use (share of time); and • Personnel time (share of salaries and benefits)
Service delivery	• Per diem payments; and • Fuel and transportation fees	• Fuel and transportation fees; • Vehicle use (share of time); and • Personnel time (share of salaries and benefits)
Supervision, monitoring and evaluation	• Per diem payments (lodging, meals)	• Vehicle use (share of time); • Cost of post-introduction evaluation; and • Personnel time (share of salaries and benefits)
Other	• National and district launch of vaccination; • Review of monitoring tools; • Stakeholders meeting; • Report-writing meeting; • Service of NGO vehicles; • Office communications; and • Networking meeting	• HPV strategic advisory group meetings; • Communications; and • Personnel time (share of salaries and benefits)

HPV: human papillomavirus; NGO: nongovernmental organization.

^a ^Financial costs were defined as actual monetary payments or expenditure by the government

^b ^Economic costs were defined as financial costs plus the value of resources already paid for or owned by government or provided by other sources (opportunity cost), including the value of donated vaccines.

## Results

The HPV vaccination project resulted in an estimated 11 599 doses delivered (11 251 at schools, 321 at health facilities and 27 at outreach points) and 5724 fully immunized girls (5540 at schools, 168 at health facilities and 16 at outreach points). The mean financial cost per dose for the overall project was US$ 19.76 and the mean economic cost per dose (which included the cost of the vaccine) was US$ 45.00 (Table 3). The mean financial and economic costs per fully immunized girl were US$ 40.03 and US$ 91.19, respectively.

**Table 3:** Introduction and recurrent costs per dose and per fully immunized girl in the Zimbabwe human papillomavirus vaccination project, 2014–2015

Cost category	Financial cost, US$				Economic cost, US$		
	Total	Per dose^a^	Per fully immunized girl^b^		Total	Per dose^a^	Per fully immunized girl^b^
Introduction costs^c^	120 981	10.43	21.14		214 637	18.50	37.50
Recurrent costs^d^	108 163	9.33	18.90		307 309	26.49	53.69
Total costs	229 144	19.76	40.03		521 946	45.00	91.19
Total costs (without vaccine and vaccine-related supplies^e^)	228 981	19.74	40.00		463 242	39.94	80.93

HPV: human papillomavirus; US$: United States dollars.

^a^ Total number of doses delivered: 11 599.

^b^ Total number of fully immunized girls: 5724.

^c^ Introduction costs are initial investments that are expected to last longer than 1 year, and include the activity categories of microplanning, training, and sensitization and information materials.

^d^ Recurrent costs are expected to last less than 1 year. This includes activity categories of vaccines (i.e. cost of vaccine, syringe and vaccine carriers and cold packs) (only included in economic cost) and vaccine-related supplies of safety boxes, and cotton (included in both financial and economic costs), service delivery, supervision, and monitoring and evaluation. Based on the WHO Cervical Cancer Prevention and Control Costing tool user guide.^18^

^e^ Vaccine procurement and injection supplies includes the procurement and distribution of vaccine, syringes, safety boxes, vaccine carriers and cold packs, and cotton. Note that for specific cost calculations, the financial cost includes safety boxes and cotton; economic cost additionally includes the cost of the vaccine including freight and transportation, vaccine carriers and cold packs, and syringes.

Note: We collected costs in current prices from 2014, 2015 and 2016, adjusted for inflation to 2016 US$ using the Zimbabwe consumer price index.^20^

The overall financial cost of the project was US$ 229 144 and the overall economic cost was US$ 521 946. Among activity categories, the share of financial cost was highest for social mobilization and information materials (24.1%; US$ 55 170) and lowest for vaccines, including vaccination-related supplies (0.1%; US$ 162; Table 4). The activity category with the highest share of economic cost was service delivery (21.7%; US$ 113 444) and the lowest was microplanning (9.6%; US$ 50 306).

**Table 4:** Share of financial and economic costs by activity in the Zimbabwe human papillomavirus vaccination project, 2014–2015

Activity	US$ (%)		
	Financial cost	Economic cost	Net economic cost (economic–financial)
Microplanning	2 619 (1.1)	50 306 (9.6)	47 687 (16.3)
Vaccine	162 (0.1)	58 704 (11.3)	58 542 (20.0)
Training	53 204 (23.2)	69 662 (13.4)	16 458 (5.6)
Social mobilization, and information materials	55 170 (24.1)	67 568 (12.9)	12 398 (4.2)
Service delivery	40 053 (17.5)	113 444 (21.7)	73 391 (25.1)
Supervision, monitoring and evaluation	35 166 (15.3)	100 611 (19.3)	65 445 (22.4)
Other^a^	42 770 (18.7)	61 651 (11.8)	18 881 (6.4)
Cold-chain supplementation	NA	NA	NA
Total	229 144 (100.0)	521 946 (100.0)	292 802 (100.0)

HPV: human papillomavirus; NA: not applicable; US$: United States dollars.

^a^ Other activity includes items relevant to introduction and recurrent cost categories that did not fit into the specific activity categories including launch of vaccination (national and district), review of monitoring tools, stakeholders meeting, report-writing meeting, HPV strategic advisory group meetings, service of nongovernmental organization vehicles, office communications and networking meeting.

Note: We collected costs in current prices from 2014, 2015 and 2016, adjusted for inflation to 2016 US$ using the Zimbabwe consumer price index.^20^

### Vaccination rounds

During the project, the mean number of girls vaccinated per school ranged between 12 and 36, which was higher than the mean number of girls vaccinated at health facilities (between 0 and 10) or outreach points (between 0 and 1; Table 5). The second vaccination round produced the largest number of doses delivered (5788; Table 6). By vaccination round, the lowest cost per dose was realized during the second round (US$ 1.97 financial cost; US$ 6.79 economic cost both in Marondera district; Table 6).

**Table 5:** Mean number of girls vaccinated in the two districts by vaccination round in the Zimbabwe human papillomavirus vaccination project, 2014–2015

Vaccination round and district	Schools				Health facilities				Outreach points		
	No. offering vaccination	No. of girls vaccinated	Mean no. of girls vaccinated		No. offering vaccination	No. of girls vaccinated	Mean no. of girls vaccinated		No. of points reached	No. of girls vaccinated	Mean no. of girls vaccinated
**First round**											
Beitbridge	69	1460	21		17	99	6		47	11	< 1
Marondera	93	1830	20		22	12	1		NA	NA	NA
**Second round**											
Beitbridge	70	2193	31		17	162	10		47	11	< 1
Marondera	95	3416	36		23	6	< 1		NA	NA	NA
**Third round**											
Beitbridge	66	762	12		10	42	4		7	5	1
Marondera	96	1590	17		23	0	0		NA	NA	NA

NA: not applicable.

**Table 6:** Service delivery costs per dose by vaccination round in two districts in the Zimbabwe human papillomavirus vaccination project, 2014–2015

Vaccination round and district	No. of doses given	Financial cost, US$		Economic cost, US$	
		Total	Per dose	Total	Per dose
**First round**					
Beitbridge	1 570	8 195	5.22	16 617	10.58
Marondera	1 842	6 440	3.50	21 761	11.81
**Second round**					
Beitbridge	2 366	6 770	2.86	16 129	6.82
Marondera	3 422	6 734	1.97	23 225	6.79
**Third round**					
Beitbridge	809	5 167	6.39	12 513	15.47
Marondera	1 590	6 746	4.24	23 198	14.59
**Total**					
Beitbridge	4 745	20 132	4.24	45 260	9.54
Marondera	6 854	19 920	2.91	68 185	9.95

NA: not applicable; US$: United States dollars.

Note: Includes service delivery activity only. We collected costs in current prices from 2014, 2015 and 2016, adjusted for inflation to 2016 US$ using the Zimbabwe consumer price index.^20^

### Service delivery costs

The service delivery costs varied by vaccination strategy. The mean service delivery costs per fully immunized girl for school vaccination, the primary strategy, were US$ 5.34 (financial cost) and US$ 17.39 (economic cost; Table 7). The mean financial and economic service delivery costs per dose for school vaccination were US$ 2.63 and US$ 8.56, respectively. In health facilities, the mean financial and economic service delivery costs per fully immunized girl were US$ 34.90 and US$ 41.25, respectively, and at outreach points were US$ 288.63 and US$ 635.84, respectively. The mean financial and economic service delivery costs per dose in health facilities were US$ 18.26 and US$ 21.59, respectively, and at outreach points were US$ 171.04 and US$ 376.79, respectively.

**Table 7:** Service delivery costs per fully immunized girl and per dose by strategy in the Zimbabwe human papillomavirus vaccination project, 2014–2015

Strategy and cost category^a^	No. of doses delivered	No. of fully immunized girls	Service delivery cost, US$					Unit cost, US$	
			Per diem payments (lodging, meals)	Personnel time (share of salaries and benefits)	Fuel and transportation fees	Vehicle use (share of time)	Total	Cost per dose^b^	Cost per fully immunized girl^b^
**School (primary strategy)**									
Financial cost	11 251	5 540	22 899	NA	6 672	NA	29 572	2.63	5.34
Economic cost			22 899	49 606	6 763	17 072	96 340	8.56	17.39
**Health facility (secondary strategy)**									
Financial cost	321	168	5 863	NA	NA	NA	5 863	18.26	34.90
Economic cost			5 863	1 016	NA	52	6 931	21.59	41.25
**Outreach (secondary strategy)**									
Financial cost	27	16	3 379	NA	1 239	NA	4 618	171.04	288.63
Economic cost			3 379	2 668	1 300	2 826	10 174	376.79	635.84
**Total (all strategies)**									
Financial cost	11 599	5 724	32 141	NA	7 911	NA	40 053	3.45	7.00
Economic cost			32 141	53 290	8 063	19 950	113 444	9.78	19.82

NA: not applicable; US$: United States dollars.

^a^ Includes service delivery activity only, excluding vaccine.

^b^ Unit cost per dose and per fully immunized girl represent an unweighted mean.

Note: We collected costs in current prices from 2014, 2015 and 2016, adjusted for inflation to 2016 US$ using the Zimbabwe consumer price index.^20^

The largest contributor to service delivery costs across all strategies was per diem payments (Table 7). These were paid to mobile teams of nurses (two to three in Beitbridge district; four in Marondera district) plus a driver, village health workers, school coordinators, command centre personnel (two in Beitbridge; three in Marondera), data collectors and one or two nurses at static health facility locations. For the school and outreach delivery strategies, per diem payments were paid to mobile vaccination teams. For the health facility delivery strategy, per diem payments were paid to health facility personnel who served as vaccinators.

## Discussion

The service delivery financial costs per dose and per fully immunized girl were consistently higher in the health facility and outreach strategies. The higher costs are probably because both strategies were primarily geared towards supporting the school vaccination strategy and because of the small number of doses delivered in these settings (348 doses; 3% of the total). There were fewer out-of-school girls than predicted and some of them were taken to be vaccinated at schools rather than via the expected health facility and outreach points. Costs for outreach and health facility strategies could be reduced if the size of mobile teams or command centre teams were reduced or if personnel were engaged without per diem payments. Any such changes in the composition of vaccination teams should be considered not only in terms of costs, but also the programmatic implications such as coverage and quality of the vaccination campaign. For school delivery, the financial cost per dose was lower when a larger number of girls was vaccinated in a single round (the second round, reaching overlapping groups of girls).

Introduction costs in this project may not be representative of national introduction costs in Zimbabwe since the country revised its plans for a three-dose vaccination series after preparations had begun, prompted by the change in WHO recommendations.^12^ To change to a two-dose schedule, information materials were re-printed, social mobilization sites were revisited and information had to be re-disseminated to communities, trainees and planning staff. This led to a higher than expected introduction cost for the project.

Zimbabwe’s implementation of a two-dose schedule delivered to two overlapping groups of girls differs from the strategies used in all previously published HPV cost studies,^13^^–^^16^ and the results are therefore not directly comparable. Nevertheless, comparison of findings from Zimbabwe with previous studies provides some context. The financial cost of service delivery per fully immunized girl in Zimbabwe was US$ 5.34 for school HPV vaccination, below the range of estimated costs published from previous HPV demonstration cost studies (from US$ 5.56 in Viet Nam to US$ 10.90 in Rwanda,^13^^,^^14^ presented in 2016 US$^21^). The economic cost of service delivery per fully immunized girl in Zimbabwe for school HPV vaccination was US$ 17.39, which is higher than the range reported in the previous HPV vaccination demonstration projects (from US$ 7.14 in Viet Nam to US$ 15.39 in Rwanda,^13^^,^^14^ presented in 2016 US$^21^). In addition to differences in country context, implementation strategy and project structure, these cost differentials reflect differences in vaccination schedule (three-dose in previous projects versus two-dose in Zimbabwe).

The findings from Zimbabwe reflect the cost to deliver HPV vaccine to two peri-urban districts and may not be directly comparable to settings represented by previous demonstration projects. For example, Zimbabwe’s economic cost per fully immunized girl was US$ 91.19, including vaccine at US$ 4.60 per dose, while in the United Republic of Tanzania the economic cost per fully immunized girl in a rural setting was estimated at US$ 115.11 (presented in 2016 US$^21^), including vaccine at US$ 5 per dose.^15^ A study of HPV vaccination projects found that as the amount of time to reach a school for vaccination increased, the cost subsequently increased.^16^ Additionally, costs could differ in contexts with less robust routine immunization systems (as measured by diphtheria–tetanus–pertussis coverage) than the two Zimbabwe project districts.

We found that the financial cost per dose decreased as the number of girls vaccinated increased. In the second round of vaccination when reaching overlapping groups of girls, the lowest financial costs per dose for vaccination service delivery were found when the most girls were vaccinated overall and on average per school. Other researchers found that the financial cost per dose decreased as the number of girls vaccinated increased;^16^ however, their analysis did not include any countries with overlapping groups or cohorts. In our study, the third round of vaccination had the highest financial cost per dose, when the fewest girls were vaccinated and the fewest average number of girls were vaccinated per school. Others reported too that service delivery costs increased as fewer girls were vaccinated per school.^16^

Our study has several limitations. The analysis was conducted retrospectively, requiring the HPV vaccination implementation staff to recall information. No written records were available for some information (e.g. personnel time); these estimates may therefore be subject to recall bias. Salary information was given by cadre, not by specific personnel involved in programme activity; therefore, the salary levels assumed may be different from actual salaries.

This project was largely executed with Gavi support and supplemental donor funding, so our results do not permit conclusions about the costs of HPV vaccination in the absence of donor support. Furthermore, our findings are based on the cost analysis of a demonstration project and do not consider underlying programmatic factors such as the performance of the routine immunization programme. Other considerations not investigated as part of our cost analysis are also important for a country’s decision on vaccination strategy. For example, the prevalence of human immunodeficiency virus (HIV) in the target population could affect decisions on HPV vaccine schedule, since a three-dose and not a two-dose schedule is recommended for people living with HIV.^6^ Furthermore, the Zimbabwe project was designed primarily to target girls through schools, with health facilities and outreach points as secondary strategies. Future evaluations of HPV vaccination projects should consider a study design allowing for comparison of effectiveness and costs of different vaccination strategies, to determine the implications for optimal use of the health facility and outreach strategies, compared with the school vaccination strategy. Finally, it is important to note that Gavi demonstration projects only represent feasibility pilots of HPV vaccination in a few districts and do not offer estimates of nationally representative costs.

Following the demonstration project, Gavi approved financial support to the government of Zimbabwe to introduce the HPV vaccine nationally.^22^ The first round of vaccination took place in May 2018 and aimed to reach 880 000 girls between the ages of 10 and 14 years (Manangazira P, personal communication, July 2018). Zimbabwe is the eighth country in Africa to introduce the HPV vaccine nationally.^22^

In conclusion, this cost analysis provides new evidence regarding the resources required to deliver a two-dose vaccination schedule of the HPV vaccine using an overlapping delivery strategy to a new target population (adolescent girls) in Zimbabwe. As part of the country’s first efforts to reach this population, the project provided an opportunity to understand the costs of various delivery strategies including health facility and outreach vaccination supplementing school-based vaccination to reach out-of-school girls. The use of health facilities and outreach points as secondary vaccination strategies likely resulted in a higher mean service delivery cost for these strategies. A lower service delivery cost per girl was found when a larger number of girls was vaccinated in each round, demonstrating scale efficiency with these larger numbers. Countries will need to reach a larger number of girls for national scale-up of the HPV vaccine, necessitating increased financial resources. Therefore, the most important lesson from this study is the potential cost savings offered by vaccinating overlapping groups.
